# Chicago Medical Colleges

**Published:** 1859-07

**Authors:** 


CHICAGO MEDICAL COLLEGES.—In our last issue we announced the resignation of Dr. Johnson, of the Chair of Physiology and Pathology, in the Rush Medical College. Since then Dr. Davis, Professor of the Principles and Practice of Medicine, and Dr. Byford, Professor of Obstetrics, have also left the school. These gentlemen have united in founding a new institution, under the name of the Lind University. The faculty consists of the following members:—Dr. Davis, Practice of Medicine; Dr. Johnson, Physiology and Histology; Dr. Andrews, Surgery; Dr. Isham, Surgical Anatomy; Drs. Rutter and Byford, Obstetrics; Dr. Mahla, Chemistry; Dr. Hollister, Descriptive Anatomy; Dr. Taylor, Pathology and Hygiene; Dr. Spafford, Medical Jurisprudence. The Chair of Materia Medica has not been filled. We have not heard of any appointments having been made to fill the vacant chairs in the Rush Medical College.

